# Assessment of COVID-19 pandemic responses in African countries: thematic synthesis of WHO intra-action review reports

**DOI:** 10.1136/bmjopen-2021-056896

**Published:** 2022-05-02

**Authors:** Ambrose Talisuna, Chinwe Iwu, J Okeibunor, Mary Stephen, Emmanuel Onuche Musa, Belinda Louise Herring, Otim Patrick Cossy Ramadan, Daniel Yota, Miriam Nanyunja, Allan Mpairwe, Freddy Mutoka Banza, Amadou Bailo Diallo, Roland Kimbi Wango, Christian Massidi, Hilary Kagume Njenge, Martin Traore, Antonio Oke, Boukare Bonkoungou, Landry Ndriko Mayigane, Ishata Nannie Conteh, Fekadu Senait, Stella Chungong, Benido Impouma, Nsenga Ngoy, Charles Shey Wiysonge, Zabulon Yoti, Abdou Salam Gueye

**Affiliations:** 1 Emergency Preparedness and Response Cluster, WHO Regional Office for Africa, Brazzaville, Congo; 2 Department of Nursing and Midwifery, Faculty of Medicine and Health Sciences, Stellenbosch University, Cape Town, Western Cape, South Africa; 3 Department of Sociology/Anthropology, University of Nigeria, Nsukka, Nigeria; 4 Emergency Preparedness and reponse Hub, WHO Regional Office for Africa, Nairobi, Kenya; 5 Disease Prevention, WHO Regional Office for Africa, Brazzaville, Congo; 6 WHE Programme, WHO Regional Office for Africa, Brazzaville, Congo; 7 Emergency Preparedness and Response, WHO Regional Office for Africa, Brazzaville, Congo; 8 Country Health Emergency Preparedness and IHR, WHO Regional Office for Africa, Brazzaville, Congo; 9 Cochrane South Africa, South African Medical Research Council, Tygerberg, South Africa; 10 Stellenbosch University, Stellenbosch, South Africa

**Keywords:** COVID-19, public health, health services administration & management, health policy, public health

## Abstract

**Objectives:**

We conducted a review of intra-action review (IAR) reports of the national response to the COVID-19 pandemic in Africa. We highlight best practices and challenges and offer perspectives for the future.

**Design:**

A thematic analysis across 10 preparedness and response domains, namely, governance, leadership, and coordination; planning and monitoring; risk communication and community engagement; surveillance, rapid response, and case investigation; infection prevention and control; case management; screening and monitoring at points of entry; national laboratory system; logistics and supply chain management; and maintaining essential health services during the COVID-19 pandemic.

**Setting:**

All countries in the WHO African Region were eligible for inclusion in the study. National IAR reports submitted by March 2021 were analysed.

**Results:**

We retrieved IAR reports from 18 African countries. The COVID-19 pandemic response in African countries has relied on many existing response systems such as laboratory systems, surveillance systems for previous outbreaks of highly infectious diseases and a logistics management information system. These best practices were backed by strong political will. The key challenges included low public confidence in governments, inadequate adherence to infection prevention and control measures, shortages of personal protective equipment, inadequate laboratory capacity, inadequate contact tracing, poor supply chain and logistics management systems, and lack of training of key personnel at national and subnational levels.

**Conclusion:**

These findings suggest that African countries’ response to the COVID-19 pandemic was prompt and may have contributed to the lower cases and deaths in the region compared with countries in other regions. The IARs demonstrate that many technical areas still require immediate improvement to guide decisions in subsequent waves or future outbreaks.

**Strengths and limitations of this study**

- Our study highlights very important findings regarding Africa’s response to the COVID-19 pandemic.
- The COVID-19 intra-action review methodology adapted by African countries in this paper is modelled after the WHO after-action review, a well-established approach used by WHO Member States across the world.
- Summarising the findings per country was difficult as the reports from countries were not homogeneous.

## Introduction

COVID-19 is caused by the novel coronavirus, SARS-CoV-2.^1^ First identified and reported from the Wuhan city of China in December 2019, this virus has spread globally and was declared a pandemic on 11 March 2020 by the WHO.^2^ Many countries were not prepared to deal with a highly infectious respiratory pathogen and were therefore caught off guard, including countries with robust health systems.^3^

The COVID-19 pandemic has continued to spread throughout the world, causing massive public health and economic disruptions. The magnitude of the COVID-19 pandemic has been lower in Africa compared with other continents.^4^ Due to the feeble healthcare system, inadequate human and financial resources in the healthcare system, and multiple existing endemic public health challenges, some experts predicted that the pandemic will be very difficult to control and will have catastrophic outcomes on the African continent.^4 5^ Initially, it was predicted that about 70 million cases and more than 3 million deaths will be recorded in Africa by June 2020.^6^ Notably, most of the models used in predictions were homogeneous in terms of the transmission dynamics of respiratory pathogens in all parts of the world including the African continent. Also, context-specific differences in terms of structural, social and environmental factors which may influence the risks of COVID-19 in Africa were not fully considered. Further, various country-led mitigation strategies were not fully integrated into the models.^7^ Nonetheless, African economies have been massively impacted on because of the pandemic and this could trigger further economic crises if not adequately contained.

African countries started preparing for the introduction of the first cases based on country connections to China.^8^ Statistical modelling predicted Egypt, Algeria and South Africa as the highest risk countries for initial introduction and spread in Africa. As predicted, Egypt was the first African country to report the first case of COVID-19^9^ while Nigeria was the first African country to report a case in sub-Saharan Africa. Luckily, Africa has previous experience in preparing for and responding to various infectious disease outbreaks, including Lassa fever, measles, cholera, Ebola virus disease, HIV/AIDS and meningitis.^8–11^ This technical know-how and experience have been rapidly adapted to the fight against COVID-19.^8^ Networks of community health agents involved in the polio eradication programme and other outbreaks have been leveraged upon for early warning at the subnational level.^12^ Investors and innovators on the continent have ramped up the domestic production of medical items such as masks, hand sanitisers, mobile applications and ventilators required to mitigate the impact of the pandemic to avoid shortages and uneven distribution.^13 14^ Research and development in terms of therapeutics and diagnostics is also active in Africa. About 33 clinical trials to assess the impact of some medical, behavioural and supportive interventions for COVID-19 were registered in Africa within 3 months of the pandemic, according to clinical trial registries.^15 16^ There is a plan to expand the capacity already in place for the next-generation sequencing to 10 more centres to improve the surveillance of the virus and further elucidate the transmission dynamics of the virus on the continent.^17^

With the number of detected new cases stagnating in some African countries and vaccines are being gradually rolled out, several countries are starting to relax their public health and social measures in an effort to resume economic activities. In accordance with recommendation from the fourth meeting of the International Health Regulation Emergency Committee for COVID-19,^18^ it is necessary for countries to review their response to the pandemic, document and share best practices. Countries also need to identify what has worked well and the challenges they have faced in the implementation of their strategic preparedness and response plans against COVID-19 so as to revise current national and subnational COVID-19 response strategies.^3^ To do this, the WHO developed a country-led COVID-19 intra-action review (IAR) process, which is aligned to the WHO COVID-19 Strategic Preparedness and Response Plan and its public health response pillars.^19^ An IAR is a country-led facilitated discussion that allows national and subnational stakeholders of the COVID-19 response to reflect on actions being undertaken to prepare for and respond to the COVID-19 outbreak at the country level, identify current best practices, gaps and lessons learnt, and propose corrective measures and actions to improve and strengthen continued COVID-19 response.^20^

In this study, we conducted a review of the available IAR reports for the COVID-19 pandemic response in Africa. We highlight the best practices and challenges, and offer perspectives for the future. The IAR findings and recommendations should be used to contribute to improved response of the COVID-19 pandemic and other concurrent public health emergencies and should ultimately lead to long-term health security.

## Methods

### Data sources and analysis

We conducted a desk review of IAR reports from countries in the WHO African Region submitted to the regional office by March 2021. These reports were mostly written based on the guidance provided by the WHO.^20^ This guidance provided countries with information regarding the purpose of the IAR, scope, format and critical domains to adapt. These domains include governance, leadership and coordination; planning and monitoring; risk communication and community engagement; surveillance, rapid response, and case investigation; infection prevention and control (IPC); case management; screening and monitoring at points of entry (PoEs); national laboratory system; logistics and supply chain management; and maintaining essential health services during the COVID-19 outbreak. The criteria for selection were mainly countries with available IAR reports. After retrieving the available reports, we summarised these reports according to these domains (online supplemental table 1).

10.1136/bmjopen-2021-056896.supp1 Supplementary data

**Figure 1:**
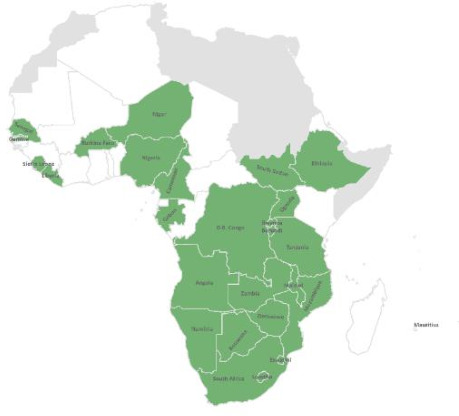
Map of African countries that had conducted and submitted their intra-action review reports by March 2021.

Twenty-five countries had conducted an IAR in the WHO Africa Region by March 2021, namely Angola, Botswana, Burkina Faso, Burundi, Cameroon, the Democratic Republic of Congo (DRC), Eswatini, Ethiopia, Gabon, Liberia, Malawi, Mauritius, Mozambique, Namibia, Niger, Nigeria, Rwanda, Senegal, Sierra Leone, South Africa, South Sudan, Tanzania, Uganda, Zambia and Zimbabwe. Of these countries, only 18 submitted their complete IAR reports to the WHO Regional Office. This study therefore provides a summary of the best practices and challenges based on these 18 countries (figure 1). We conducted a thematic analysis using manual coding.

The IAR reports are not yet in the public domain and were retrieved from the WHO Regional Office for Africa by the authors (AT and JO). CI and CSW conducted the thematic analyses of the reports.

### Patient and public involvement

The study presents analysis of secondary data. There was no patient or public involvement.

## Results

Eighteen IARs were assessed in this study. These reports were not yet publicly available at the time of writing this paper. Online supplemental table 1 summarises the best practices, challenges and recommendations provided by the countries in these IARs, according to the 10 domains of the COVID-19 preparedness and response pillars. We could not summarise these findings per country because the reports from countries were not homogeneous. For example, not all countries adhered strictly to the guidance for conducting IAR and the reporting format proposed therein. Even with those that followed the guidelines, reporting was not uniform, since countries had their peculiar best practices and challenges. We, therefore, summarised the key points that were common among the reports reviewed. These points are therefore not peculiar to all countries. Subsequently, we provide a synthesis of the key lessons learnt.

### Governance, leadership and coordination

The countries’ key best practices include strong leadership as evidenced by the prompt activation of national technical multisectoral response structures and political will prompting sensitisation across various media, including debunking of misinformation. Most countries established national committees chaired by the president or prime minister before the first cases were imported into the countries. In addition, countries leveraged on existing health coordination structures and surveillance systems, including using influenza sentinel surveillance sites. However, a common challenge noted was the difficulty in striking a balance between sharing accurate information and earning public trust, considering the overwhelming rumours and misinformation on various social media platforms. Other challenges included shortages of experienced health workers to fully manage emergency preparedness, lack of established Emergency Operation Centres (EOCs) which resulted in poor definition of Incident Management Systems (IMS), delays in development and dissemination of guidelines, and regular review and update of guidelines.

### Surveillance, rapid response and case investigation

The role of the Integrated Disease Surveillance and response System (IDSR) in detection of index cases was key in most countries. The events-based surveillance arms were critical in picking up rumours and facilitating prompt investigation. Also, case definitions for COVID-19 were quickly incorporated into the IDSR reporting in most countries. Initially, there was timely case investigation coupled with timely contact tracing. However, as case numbers increased, case investigation and contact tracing became suboptimal. Similarly, there was screening surveillance at various (PoEs). Modelling studies were conducted to generate predictions that enabled the scaling up of testing and case management capacities. In addition, healthcare workers (HCWs) were trained on surveillance standard operating procedures (SOPs), tools and guidelines. Some countries deployed field epidemiologists to assist with collation, analysis, reporting, contact tracing and investigation of new clusters. These included Ethiopia, South Africa, Mozambique, Kenya, DRC, Uganda, Zambia and Zimbabwe. Community health workers were also mobilised to conduct contact tracing and active case searches. There was also the incorporation of digital contact tracing to enhance the process in Rwanda and South Africa. The challenges encountered under this domain include the difficulty in managing case investigation as the case numbers increased in most countries, including difficulties in locating contacts due to wrong addresses and resistance from communities. Further, there were concealment of symptoms by travellers or incoming passengers, including potential cases within the communities which made contact tracing difficult.

### Screening and monitoring PoEs

There was early closure of borders, compulsory quarantine for travellers, comprehensive screening and travel bans for high-risk countries. Furthermore, information education and communication materials were made available at PoEs, including thermal scanners and infrared thermometers. The staff involved in these processes were trained. PCR tests, and later rapid antigen tests, were conducted for inbound and outbound travellers. Although structures were put in place for quarantine and IPC measures, countries were not prepared for the high influx of returning citizens that in some countries overwhelmed border control personnel. This lack of preparation led to inadequate isolation facilities and shortages of personal protective equipment (PPE), coupled with lack of human resources and inadequate communication at some PoEs.

### Infection prevention and control

There were immediate and stringent lockdowns imposed following the detection of the index case. This was backed by provision of PPE, creation of quarantine centres, testing and isolation of persons who tested positive. The WHO immediately provided IPC guidelines. Furthermore, during the initial days of lockdowns in countries, work access permits were granted to essential workers to control the movements of citizens. However, poor adherence to and enforcement of IPC measures was reported in several countries. Shortages of PPE occurred when the numbers of cases spiralled in most countries. The shortages were further worsened by irrational use. In some instances, there was inadequate supply of water in health facilities, which compromised IPC measures. Indiscriminate disposal of surgical masks was also reported in various countries.

### Case management

With regard to case management, prompt deployment of experts, training, guidelines and SOPs were made available to guide clinical management of COVID-19 in most countries. Some countries like Mauritius, Nigeria and South Africa introduced a community case management model to reduce pressure on the health system. There was systematic isolation of persons who were infected, including asymptomatic patients. However, there was lack of specialised infectious disease hospitals and inadequate technical expertise to manage severe cases of COVID-19 in many countries. The constant change of treatment protocols also contributed to these challenges. Key challenges in case management were shortages of oxygen delivery capacity, inadequate ventilators and inadequate intensive care unit (ICU) and high dependency unit (HDU) capacity.

### National laboratory system

There was an early development of testing capacity enabling the detection of imported cases. Testing capacity was further boosted through the rapid roll-out of mobile testing and involvement of the private sector in several countries. The pandemic influenza surveillance programme and national influenza centres, and highly skilled personnel were also an added advantage in several countries. Another notable best practice was introducing a logistics management information system for data storage and dissemination, as seen in Republic of South Sudan. However, testing was impeded by the global shortages of laboratory reagents to meet the growing demand. In addition, testing capacity was inadequate in most countries and this was further impacted when the laboratory personnel became infected with the virus, with some suffering burnouts due to excessive work.

### Risk communication and community engagement

There were daily updates of the number of cases as the pandemic progressed in all countries to build trust in the people. There was development of communication materials for different targeted groups and dissemination through multiple channels and readjusting of the messages based on the emerging issues. The challenges include a reactive rather than a proactive communication, especially as it relates to mainstream and social media misinformation, as reported by South Africa. Further, lack of resources delayed communication.

### Maintaining essential health services during the COVID-19 outbreak

Essential services such as provision of antiretroviral therapy for HIV and maternal and child health services were greatly impacted in most countries, with limited availability of essential medicines, vaccines and medical supplies. Countries such as South Africa provided guidelines for the provision of these services, and IPC measures were adhered to during the delivery of these essential services. Malawi suffered disruption of essential services. There was also interruption of immunisation campaigns in the early days of the pandemic. In addition, a reduction of uptake of essential services due to the fear of getting infected at the facilities was noted in Malawi and Botswana. With the easing of lockdowns, there was an increase in attendance in facilities for these services in most countries.

## Discussion

This study highlights several best practices, but there were also major challenges that need to be addressed moving forward in African countries for better COVID-19 response. From the review, it can be deduced that the initial COVID-19 response in the 18 countries included in this analysis was commendable. Further, the uptake of the IAR process has been very high in the WHO African Region—the IARs conducted in the African region constitute about 80% of all IARs conducted globally. There was strong leadership and political will in almost all countries. Countries also leveraged, to their advantage, already available systems, such as surveillance systems for infectious diseases like Ebola, polio, influenza, cholera, tuberculosis and HIV.^21 22^ The timely response was likely due to the experience from the 2014–2016 Ebola virus disease outbreak in West Africa. African leaders saw the need to respond swiftly since failure to contain COVID-19 would threaten health, prosperity and security.^8^ Initially, African countries were focused on preventing the importation of COVID-19 and containing onward transmission within countries. As early as January 2020, many African countries started implementing enhanced surveillance at PoEs, screening all passengers for a recent history of travel to China and screening for fever.^8^ Another area of swift response was noted when Africa started to prepare for its first cases that would arise from its close connections from China. Egypt, Algeria and South Africa were identified through modelling techniques to be the countries likely to be among the first to introduce and spread the virus on the continent.^8^ This swift response to COVID-19, born of Africa’s experience of dealing with constant outbreaks of infectious diseases in the past, probably explains the comparatively low COVID-19 cases reported from Africa. However, it is also possible that other factors such as limited clinical surveillance and inadequate laboratory facilities may have contributed to the lower numbers of cases reported from the continent.^23^

One year into the response, one of the common challenges faced is fatigue of the response teams. In some countries, the IMS structures have not been linked to the EOCs, while in others the IMS operated without any linkage to any EOCs. Therefore, the IMS structures have been rigid and not scalable, resulting in fatigue and burnout among the IMS teams. Moving forward, the IMS teams need to be linked to EOCs and aligned to the resurgence thresholds so that at control level the IMS can be scaled down to core functions and the frequency of meetings reduced so that key personnel get time to rest. As resurgence alert levels are reached, the IMS can be progressively scaled up. The operationalisation of this core principle of the IMS will also allow for other essential health services to be remedied at low levels of transmission as some repurposed staff can revert to their routine jobs. The challenges encountered mostly arose when the number of cases increased significantly thereby affecting the response capacity at various levels. This is a key lesson that will inform decision-making in subsequent waves or future outbreaks.

COVID-19 is a new disease, and this impacted on case management as noted in this review. This was confirmed by an assessment conducted by Umviligihozo *et al.*^21^ The majority of African countries lack specialised medical capacity required for handling severe cases of COVID-19, including ICU beds. Before vaccines were developed, no effective treatments were available, and many countries had to rely on the evolution of evidence of several treatment protocols. This also was responsible for the ever-changing guidelines for case management. COVID-19 being a new disease may have also impacted on the ability of communities and health workers to adequately adhere to stringent IPC measures. Countries are therefore encouraged to keep up with their best practices by further strengthening them while developing interventions for those areas requiring improvement. For example, good attention should be paid towards training personnel involved at various levels of the COVID-19 response, from the PoEs to health facilities and the communities. The laboratory capacities and quarantine facilities should also be improved.

Based on the challenges identified, and the common recommendations presented by different countries, we offer the following perspectives for the future. Regarding governance, coordination and planning, there is an urgent need to improve coordination at all levels—continental (regional), national, but more importantly, at subnational level. All countries should develop and finance emergency preparedness plans for future outbreaks. Further, there is a need for a central repository for public health strategies that is accessible to all programme managers at all levels. With respect to risk communication and community engagement, SOPs are needed that will guide risk communication strategies. Community leaders and COVID-19 survivors can be useful champions to sensitise the public on the realities of the pandemic. There is a need to support countries to invest in preparedness for future outbreaks. A robust integrated surveillance system is needed in all countries and there is a need to scale up the use of digital innovations to increase timeliness of detection and response. Countries are also urged to periodically conduct COVID-19 prevalence studies to help guide decision-making.

Countries need to provide adequate supplies of PPE and digital screening tools at PoEs. Timely information-sharing between neighbouring countries is very important. Adequate human resources should be provided at PoEs. All countries should develop and implement public health emergency contingency plans for various PoEs like airports, seaports and major land borders.

IPC strategies should be reviewed across all health programmes based on clearly defined roles, reporting and accountability across national and subnational levels. Moreover, IPC training should be implemented to ensure health personnel adhere to standard IPC practices. In areas where there is little or no water supply, clean and adequate supply of water should be provided. Health promotion within the communities should be strengthened to enhance adherence to IPC measures within communities. There is also a need for a functional IPC programme in hospitals and primary health centres that is well maintained and tested regularly during peace time. Importantly, there is a need for continuous training of health workers and availability of PPE to prevent health worker infections.

With respect to case management, there is a need for regular updates and dissemination of case management guidelines as new information becomes available. In addition, there is an urgent need for information-sharing between ministries of health and the private sector on the management of COVID-19. HCWs should undergo training on case management of COVID-19. In the long-term, case management of COVID-19 should be integrated into continuity of services. Further, countries should urgently increase their capacity for critical care of patients at national and subnational levels in terms of availability of trained health workers, availability of oxygen concentrators, oxygen plants, ventilators, and ICU and HDU beds. Furthermore, countries should set up flexible mechanisms for the quick mobilisation of trained HCWs in case there is a need for surge capacity for case management and mechanisms to quickly establish community treatment centres in a short time, including surge for supplies, equipment and personnel, drawing lessons from the devastating effects of new waves of the COVID-19 pandemic being observed.

Given the weakness of national laboratory systems in various countries, there is a need for the scale up of testing capacities coupled with the establishment of more regional laboratories. In addition, it is important to ensure pre-stocking of consumables and reagents in preparation for subsequent wave(s) of the COVID-19 pandemic. Further, training on inventory management of consumables is needed and the rational use of PPE is highly recommended.

Regarding logistics and the supply chain, African countries need to subscribe to a pooled procurement mechanism for essential and emergency products using global pool procurement facilities. In addition, there is a need for skilled human resources to be recruited for data management and logistics. Demand forecasting is needed, and HCWs should be trained on forecasting and general inventory management. Importantly, the local manufacture of PPE is highly recommended.

African countries should use lessons learnt from COVID-19 development plans to build resilient health systems that can ensure continuity in the provision of essential services during emergency situations. In addition, a robust system for monitoring the provision of these services is needed as well as a rapid assessment of the impact of the outbreak on the uptake of these services.

The COVID-19 IAR methodology used by countries in this paper is modelled after the WHO after-action review (AAR), which is a well-established approach used by WHO Member States across the world, including in Africa. The AAR is a component of the International Health Regulations Monitoring and Evaluation Framework, which aims to assess the functional capacity of public health and emergency response systems and to identify practical areas for continued improvement. According to the WHO AAR guidance published in 2019, AARs should be conducted within 3 months after the emergency event.^24^

Despite the important findings from this review, it was not devoid of limitations. Considering the heterogeneity in the reports submitted by countries, we conducted a thematic analysis of the key areas common among countries. There is a possibility that with such an approach, we may have missed important lessons and challenges unique to certain countries. That notwithstanding, our study shows that experience from previous disease outbreaks meant that most African countries were in a state of preparedness that helped them to respond appropriately to COVID-19. Stakeholders are advised to identify areas that apply more to them to guide improvement processes in their countries.

## Conclusions

The findings of this study suggest that African countries’ response to the COVID-19 pandemic was timely and contributed to the lower cases and deaths due to COVID-19 reported on the continent compared with other regions. Nevertheless, many areas still require improvement, and these areas need immediate attention to guide decision-making during subsequent waves of the COVID-19 pandemic. If corrective measures are not immediately taken, future waves of the pandemic could overwhelm the weak health systems in Africa and lead to disastrous consequences.

## Data Availability

Data are available upon reasonable request. Access to the IAR reports for individual countries will be facilitated upon request to the corresponding author.

## References

[1] Du Toit A. Outbreak of a novel coronavirus. Nat Rev Microbiol 2020;18:123. 10.1038/s41579-020-0332-0 31988490 PMC7073251

[2] WHO Director-General’s opening remarks at the media briefing on COVID-19 - 11 March 2020. Available: https://www.who.int/director-general/speeches/detail/who-director-general-s-opening-remarks-at-the-media-briefing-on-covid-19-11-march-2020 [Accessed 2 May 2021].

[3] Mayigane LN, de Vázquez CC, Vente C, et al. The necessity for intra-action reviews during the COVID-19 pandemic. Lancet Glob Health 2020;8:e1451–2. 10.1016/S2214-109X(20)30414-9 33038949 PMC7544463

[4] Maeda JM, Nkengasong JN. The puzzle of the COVID-19 pandemic in Africa. Science 2021;371:27–8. 10.1126/science.abf8832 33384364

[5] World Economic Forum. Why sub-Saharan Africa needs a unique response to COVID-19, 2020.

[6] Walker PGT, Whittaker C, Watson OJ, et al. The impact of COVID-19 and strategies for mitigation and suppression in low- and middle-income countries. Science 2020;369:413–22. 10.1126/science.abc0035 32532802 PMC7292504

[7] Twahirwa Rwema JO, Diouf D, Phaswana-Mafuya N, et al. COVID-19 across Africa: epidemiologic heterogeneity and necessity of Contextually relevant transmission models and intervention strategies. Ann Intern Med 2020;173:752–3. 10.7326/M20-2628 32551812 PMC7384264

[8] Massinga Loembé M, Tshangela A, Salyer SJ, et al. COVID-19 in Africa: the spread and response. Nat Med 2020;26:999–1003. 10.1038/s41591-020-0961-x 32528154

[9] Nkengasong J. Let Africa into the market for COVID-19 diagnostics. Nature 2020;580:565. 10.1038/d41586-020-01265-0 32346145

[10] World health organisation (WHO). Regional office for Africa. strategic response plan for the who African region of contents, 2019.

[11] Afolabi MO, Folayan MO, Munung NS, et al. Lessons from the Ebola epidemics and their applications for COVID-19 pandemic response in sub-Saharan Africa. Dev World Bioeth 2021;21:25–30. 10.1111/dewb.12275 32654261 PMC7404531

[12] WHO. Nigeria’s polio community health agents take on COVID-19 detection. WHO, Regional Office for Africa, 2020.

[13] Setipa J. Help developing countries to manufacture their own medical equipment to solve supply shortages, 2020.

[14] Nebe C, Jalloh A. Coronavirus pandemic driving Tech solutions in sub-Saharan Africa. Africa: DW, 2020.

[15] Africa CDC. Outbreak brief 18: COVID-19 pandemic. Africa CDC, 2020.

[16] Africa CDC. COVID-19 scientific and public health policy update – 19 may 2020 –. Africa CDC, 2020.

[17] Africa CDC. Illumina partners with Africa CDC to strengthen sequencing capacity for COVID-19 surveillance in Africa. Africa CDC, 2020.

[18] World Health Organisation. Statement on the fourth meeting of the International health regulations (2005) emergency Committee regarding the outbreak of coronavirus disease (COVID-19. Geneva, Switz: WHO, 2020. https://www.who.int/news/item/01-08-2020-statement-on-the-fourth-meeting-of-the-international-health-regulations-(2005)-emergency-committee-regarding-the-outbreak-of-coronavirus-disease-(covid-19)

[19] WHO. Monitoring and evaluation framework. COVID-19 strategic preparedness and response, 2020.

[20] World health organisation (WHO). Guidance for conducting a country COVID-19 intra-action review (IAR), 2020. Available: https://www.who.int/publications/i/item/WHO-2019-nCoV-Country_IAR-2020.1

[21] Umviligihozo G, Mupfumi L, Sonela N, et al. Sub-Saharan Africa preparedness and response to the COVID-19 pandemic: a perspective of early career African scientists. Wellcome Open Res 2020;5:163–22. 10.12688/wellcomeopenres.16070.2 32984549 PMC7499400

[22] Dzinamarira T, Dzobo M, Chitungo I. COVID-19: a perspective on Africa's capacity and response. J Med Virol 2020;92:2465–72. 10.1002/jmv.26159 32525568 PMC7300956

[23] Senthilingam M. Covid-19: why Africa's pandemic is different. BMJ 2021;375:n2512. 10.1136/bmj.n2512 34667031

[24] Guidance for conducting a country COVID-19 intra-action review (IAR). Available: https://www.who.int/publications/i/item/WHO-2019-nCoV-Country_IAR-2020.1 [Accessed 10 Apr 2021].

