# Supplementary material for: Assessment of COVID-19 pandemic responses in African countries: thematic synthesis of WHO intra-action review reports
Source: BMJ Open. 2022 May 2;12(5):e056896. doi: 10.1136/bmjopen-2021-056896 (PMC9062458; doi:10.1136/bmjopen-2021-056896)
Supplement: Supplementary data [file bmjopen-2021-056896supp001.pdf]

Table 1: Summary of challenges and best practices documented in 18 intra-action review reports available by March 2021 in Africa

	Domain	Challenges	Best Practices
1	Coordination, Planning, and Monitoring	Gaps in the implementing preparedness and response plan to COVID-19: Botswana	Early assessment and strengthening of preparedness and response capacities in Botswana
		Testing of the preparedness plan at outbreak onset not done through drills or simulation exercises	Clear leadership and strong political commitment in COVID-19 response with the creation of a high-Level committees on COVID-19 pandemic control: Botswana.
		Some provinces/States do not have fully operational IMTs and do not coordinate effectively with the districts/councils, impeding the pace of implementing key interventions	Early activation of the Incident Management System (IMT) and creation of an inter-sectoral committee (technical pillars)
		Forecasting did not address some important aspects of the response e.g. surge capacity, financial resources	Evolving preparedness and response plans adapted to the ever-changing global and local context and WHO guidelines: Botswana
		Piece-meal release of guidelines which were disseminated in soft copy but not easily accessible to all	Decision making at technical and policy level are in synchrony
		Inadequate funds for COVID-19 preparedness measures following onset	Regional and sub-regional collaboration via weekly meeting for cooperation and sharing of best practices
			Government embracing partnerships and support from bilateral and multilateral agencies, private sector, foundations, and friendly countries<br><br>Inclusion of COVID-19 Responses in Health Sector Strategic Plan 2020-2024
2	Risk Communication and Community Engagement	Diffusion of personal information on patients in the media and on social media platforms	Early intense sensitization campaign on COVID-19 by the Ministry of Health across wide range of media with the close collaboration of key stakeholders for maximum reach: Botswana

		Difficulty in striking the right balance between sharing accurate and reliable information and stressing on public health measures put in place without creating fear and panic among the population	All official diffusion of information from the Government emanated from the National Presidential Task Force (or Nat'l Communication Committee) on COVID-19 via the daily press conference, resulting in direct and harmonized dissemination of information: Botswana
			Diverse platforms for diffusion of communication such as the hotline, daily press conferences, facebook, websites and mobile application, for maximum reach and meeting the varied information seeking behaviours of the different segments of the population
		Rapidly spreading rumours and 'fake news' which were easily accepted by the population: Botswana	Fake news on social media was sanctionable to dissuade the spreading of misinformation
		Limited financial resources delayed relaying communication through local radio stations	Media programmes and IEC material produced in local languages and braille, especially in Zambia and South Africa.
3.	Surveillance, Rapid Response, and Case Investigation	Delays in the contact tracing exercise due to the escalating number of positive cases as the outbreak evolves	Relatively efficient and prompt case investigation and scaled up contact tracing in Bostwana,
		Difficulty in locating contacts due to inaccurate addresses slowed down the contact tracing exercise	Early attention to surveillance at points of entry with double temperature checks and specific investigation followed by community surveillance by health inspectors for 14 days
		Resistance to self-isolation for asymptomatic contacts	Multiple surveillance systems were used: surveillance at borders, community surveillance, hospital and laboratory surveillance and the sentinel surveillance system for influenza-like illness (ILI) and Severe Acute Respiratory Infection Surveillance (SARI).
		Difficulty in ascertaining that contacts are abiding by public health instructions	Establishment of modelling team/consortium generating predictions from the models guiding the scaling up of case management and testing capacities. Example is the South African COVID-19 Modelling Consortium. (Ethiopia was al

		Resistance to isolation/monitoring in treatment centre, especially for positive asymptomatic contacts	Daily, weekly reporting and provision of feedback enabled tracking of the trajectory of the epidemic (All countries)
		Responding to multiple outbreak and humanitarian issues. For example, Dengue in Mauritius	Deployment of field epidemiologists & surveillance officers (from government and WHO) who assisted with collation, analysis, reporting, contact tracing and investigation of new clusters
		High risk of concealment of symptoms among travelers or incoming passengers or potential cases within communities	Coordination with different stakeholders at the local level (e.g. NGOs, academia, private sector) brought onboard additional expertise that improved data analysis, case investigation and contact tracing. E.g Botswana
		Knowledge gaps particularly around the level of herd immunity, duration of immunity, correlates of protection delayed provision of guidance and the design of interventions	Use of digital contact tracing applications like COVID-Connect
		Lack of community support for contact tracing	Use of CHW as a backbone to contact tracing
		Increased caseload and wide geographical spread constrained the available resources in a short time	Repurposing existing staff for case investigation and contact tracing
4	Points of Entry	Lack of preparedness for unprecedented border closure and the need to rapidly scale up quarantine capacities and Infection Prevention and Control (IPC) measures to welcome the high volume of incoming citizens in the last flights	Early quarantine measures for incoming passengers with high-risk characteristics
		Lack of ICT connectivity for remote PoE	Comprehensive screening of all travellers while still aboard conveyances
		Lack of isolation facilities in some PoE	Free compulsory quarantine for all returning citizens. All countries
		Inadequate supplies, equipment and other logistics at PoE e.g, PPEs, gloves, swabs, airtime, data bundles, tablets and transport	Rapid closure of borders upon detection of the first few cases of COVID-19, all of which were imported cases. All countries

		Economic toll of closed borders due to heavy reliance on both tourism and import and export services and close links to Asia and Europe	Prompt imposition of travel restrictions for high-risk countries
		Incidences of disturbance in quarantine centre from individuals contesting the restriction in spite of lengthy and repeated sensitisation	Amended testing strategy for quarantine from the only exit test to regular testing on Day 0, Day 7 and Day 14 for early detection and isolation of cases and reduced costs of quarantine
		Inadequate risk communication at PoEs	Traing and Engagement of additional staff to address the shortage of personnel at the PoE. All countries
		Lack of signage to guide travellers on processes	Availability of partner support (AFENET, WHO) towards monitoring of travellers on quarantine using mobile phone
		Human resource deficiency	Timely provision of Information, Education and Communication (IEC) materials at PoE
		Insufficient collaboration between private and public sector at entry point level	Availability of data collection tools at PoE
		Lack of coordination and harmonization of data among different PoE (aerial, land and maritime)	Capacity building on surveillance, IPC and data management to POE staff. Botswana
		Poor knowledge of viral ecology with human and environment interface	Leveraging on existing cross-border collaborations with neighbouring countries
		Difficulties in implementation of legislation and lengthy processes in the review of the legislative framework	Availability of thermal scanners and hand-held infra-red thermometers at PoE
			Suspension of cruise ships and passenger ships e.g. Senegal<br>PCR testing for in- and outbound travellers<br>Setting up of sanitary protocols for incoming cattle and their breeders during Tabaski for early detection, as seen in Senegal<br>Decentralisation of diagnostics to the field enhanced early detection of case (reagent and GeneXpert deployed) as seen in Senegal

5	Infection and Prevention Control	Initial challenges in implementing IPC measures in healthcare settings with limited availability of the PPE and health personnel poorly adopting the new sanitary measures	Immediate and stringent national lockdown following the detection of the first few cases to halt the spread of COVID-19 and allow for the detection of positive cases via contact tracing
		Inconsistent application of IPC measures leading to varying degrees of health care worker infection across countries	Strict IPC measures implemented for the safety of the health care workers such as provision of adequate PPE, isolation during their working shifts with provision of accommodation, quarantining and testing at the end of their work shifts
		IPC planning and preparation for the pandemic started late and delayed training and implementation	Availability of national IPC training guideline and step-down of training supported by WHO. All countries
		Initial poor management and irrational use of PPE leading to inadequacies in stock	The creation/amendment and implementation of legal frameworks to prevent the spreading of COVID-19 to reinforce guidelines for IPC measures
		Poor coordination of trainings between MoH and partners resulting in duplication of efforts, insufficient district coverage and incomplete practical demonstrations	Adoption of IPC measures across all sectors of the whole-of-government approach facilitated by the High-Level Committee on COVID-19 and different multisectoral initiative
		Poor enforcement of public health measures by relevant authorities	Development of tools for monitoring IPC in PoE and facilities
		Low risk perception by the public on COVID-19 led to poor compliance to IPC measures	Working with local authorities and local leaderships in fostering compliance
		Indiscriminate disposal of surgical masks in the community	Introduction of the Work Access Permit for workers on essentials services to facilitate the monitoring of the movement of citizens during lockdown
		Poor waste segregation and color coding in the health facilities	Immediate and stringent national lockdown following the detection of the first few cases to halt the spread of COVID-19 and allow for the detection of positive cases via contact tracing
		Inadequate water supply for IPC in some facilities	Strict IPC measures implemented for the safety of the health care workers such as provision of adequate PPE, isolation during their working shifts with provision of

			accommodation, quarantining and testing at the end of their work shifts
6	Case Management	Lack of specialized infectious disease hospitals with modern hi-tech facilities and bed capacity	Guidelines and SOPs developed on case management available to guide uniform clinical management
		Countries with relatively high prevalence of NCDs have a relatively higher mortality associated with COVID-19	Introduction of the community case management model reduced the pressure on the health system
		Sub-optimal data management including limited integration from different sources	Systematic isolation of all persons infected with SARS-CoV-2 in treatment centres, including asymptomatic patients with free treatment offered
		Seeking palliatives for the family member of the COVID-19 victim was daunting financially for government to sustain	Knowledge exchange with international experts on latest updates about testing and treatment of COVID-19 and offering of the best treatment and care
		Lack of financial support given to vulnerable groups affected with COVID-19, this resulted into resistance to facility isolation or failure to observe public health guidelines	Clinicians Training Team on Case Management established to provide training support on guideline implementation and information dissemination
		Limited supplies for case management	
		Inadequate technical expertise to manage severe cases	
		Problem of health care worker infection impacting on commitment of health workers and stigmatisation	
		Trainings initially funded by government did not incorporate private sector	
		Rapidly changing case management protocol	
		Delay in results also impacted negatively on case management	

7	National Laboratory System	Inadequate coordination, prioritization, consultation and capacity for COVID-19 testing strategies	Early development of the testing capacity for SARS-CoV-2 enabled the prompt detection of the imported cases
		Inadequate incentives provided for staff	Rapid roll-out of mobile testing improved access to testing and reducing the turnaround time
		Missing variables on laboratory forms and some samples not accompanied by forms led to time lost sorting samples	Collaboration with the private laboratories significantly boosted the testing capacity across the country
		Delays in processing samples from BIDs and in-patients. E.g Zambia	All laboratories were subjected to quality assurance tests before being accredited as SARS-CoV-2 testing laboratories
		Lack of in-country capacity (engineers) to service and maintain equipment. E.g Zambia	Existence of pandemic influenza surveillance program (PIP), national influenza centres (NIC) and viral haemorrhagic fever (VHF) laboratories
		Inadequate laboratory reagents and supplies that could sustain the demand due to limited market availability of supplies	Availability of competent personnel, skills and supplies for the start-up process
		Management of laboratory resources without compromising the quality standards for testing for other pathologies and biological/clinical tests while facing an increased demand for COVID-19 testing	Joint partnership between government and development partners in procurement of supplies
		Cases of infection of technicians and contamination as well as burn-out of lab personnel as seen in Zambia	Epidemiologic numbers used within the laboratory network are shared which helped a quick return of results (and data harmonization)
		Shortage of consumables and reagents for testing at the beginning of the outbreak with the rapid escalation of positive cases and increased demand for testing. E.g Zambia	Central reporting, data management through the confidentiality and data declaration as well as mandatory sharing of data for surveillance and bio-banking
		Difficulty in purchasing consumables and reagents, due to high global demands and competition among countries and relatively small orders compared to other countries	Expanding the testing capacities of the central health laboratory via recruitment of additional technicians, 24/7 operating and acquisition of additional laboratory equipment

		Lack of cooler boxes for the RRTs due to stacks of unprocessed samples	Introduction of the Laboratory information management system (LIMS) for COVID-19 during the outbreak to improve the management, access and storage of key data and facilitate quick dissemination of results
		The bulk of laboratory testing has come from the private sector, this presented huge coordination challenges in South Africa	Mitigation of biosafety risks with intensified safety measures such as increase in number of biosafety cabinets, staff training and wearing personal protective equipment (PPE)
8	Operational support and logistics in the management of supply chains and the workforce	Shortages of PPEs for health care workers and frontline workers<br>Product misuse and wastages<br>Problems with quantification of stocks<br>Lack of locally manufactured products hence the heavy reliance on importation of medical products<br>Lack of emergency procurement guideline<br>Delays in delivery of products	Existing emergency preparedness and response plan for Ebola in countries like Malawi, Rwanda, and South Sudan<br>Engaging private sectors and NGOs for logistics support, as seen in Rwanda<br>Integrated supply chain system<br>Emergency procurement for timely delivery of essential medical materials<br>Already existing operational support and logistics in the management of supply chains and the workforce. In South Africa for example leveraged on stock visibility systems to facilitate tracking of stock levels
9	Maintaining essential health services during COVID-19 outbreak	Absence of Essential Health Services pillar on the IMS structure (initially)	Continued active surveillance for other reportable diseases using IDSR
		Absence of a plan for continuity of essential health services during outbreaks/public health events	HIV/ART care and Maternal and Child health services continued, including implementation of child health week activities, during the outbreak
		In the early stages of the outbreak, there was a reduction in the number of people accessing EHS due to fears of getting infected	Availability of guidelines for the provision of essential health services
		Inadequate focus and evaluation of essential health services provision during the COVID-19 response	Monitoring and evaluation continued sharing trends on various indicators
		Consequent backlog in elective care and increased attendance (post lockdown) since resumption of services	Consistent release of funds for essential services by government and partners

		Difficulties in organising treatment abroad for patients needing specialised surgery	Implementation of IPC measures to protect inpatients and outpatients while maintaining essential services
		Difficulties in detecting the scale of the dengue outbreak due to decrease in healthcare facilities' functionality	Maintaining and scaling-up of the yearly anti-influenza vaccination campaign (in Mauritius)
		Risk of increase incidence of Hepatitis C and HIV due to the observed increase in risky behaviours among people who inject drugs caused by the disruption of needle and syringe programmes (in Mauritius)	Maintained essential health services and the introduction of teleconsultation in the private health sector
		Interrupted immunisation vaccination campaigns for infants, children and adolescents presented a risk of reversing the past achievements of other communicable diseases (in Mauritius)	Stock evaluation and ensuring the availability of essential medications, equipment and supplies for diseases and life course conditions other than COVID-19.
		Disrupted public health services in Rodrigues despite no registered cases of COVID-19 on the island (in Mauritius)	
